# Detection of Ticks and Tick-Borne Pathogens of Urban Stray Dogs in South Africa

**DOI:** 10.3390/pathogens11080862

**Published:** 2022-07-30

**Authors:** Clara-Lee van Wyk, Khethiwe Mtshali, Moeti O. Taioe, Stallone Terera, Deon Bakkes, Tsepo Ramatla, Xuenan Xuan, Oriel Thekisoe

**Affiliations:** 1 Unit for Environmental Sciences and Management, North-West University, Potchefstroom 2520, South Africa; claraleevanwyk@gmail.com (C.-L.v.W.); TaioeM@arc.agric.za (M.O.T.); 21205450@nwu.ac.za (T.R.); thekisoe@gmail.com (O.T.); 2 Department of Biomedical Sciences, Tshwane University of Technology, Arcadia Campus, Pretoria 0001, South Africa; kmtshali@yahoo.com; 3 Epidemiology, Parasites and Vectors, Agricultural Research Council, Onderstepoort Veterinary Research, Onderstepoort 0110, South Africa; 4 Potchefstroom Animal Welfare Society, Potchefstroom 2531, South Africa; stalloneterera@gmail.com; 5 Gertrud Theiler Tick Museum, Agricultural Research Council, Onderstepoort Veterinary Research, Onderstepoort 0110, South Africa; BakkesD@agric.za; 6 National Research Center for Protozoan Diseases, Obihiro University of Agriculture and Veterinary Medicine, Obihiro 080-8555, Japan

**Keywords:** *Anaplasma phagocytophilum*, *Babesia* spp., *Coxiella* spp., *Rickettsia* spp., *Ehrlichia canis*, *Haemaphysalis elliptica*, *Rhipicephalus sanguineus*

## Abstract

This study aimed to identify ticks infesting dogs admitted to the Potchefstroom Animal Welfare Society (PAWS) and to detect tick-borne pathogens they are harbouring. A total of 592 ticks were collected from 61 stray dogs admitted to PAWS originating from several suburbs in and near Potchefstroom, South Africa. The dog ticks were identified as *Haemaphysalis elliptica* (39%) and *Rhipicephalus sanguineus* (61%) by both morphological and DNA analyses. Of these ticks, *H. elliptica* consisted of 67.5% (156/231) and 32.5% (75/231) female and male ticks, respectively, whilst *R. sanguineus* consisted of 48.5% (175/361) and 51.5% (186/361) female and male ticks, respectively. Microscopic examination of blood smears from engorged female ticks indicated overall occurrences of 0.5% (1/204) for *Babesia* spp. from *R. sanguineus*, 1% (2/204) of *Anaplasma* spp. from *H. elliptica*, and 22% (45/204) of *Rickettsia* spp. from both *H. elliptica* and *R. sanguineus.* Using pooled samples molecular detection of tick-borne pathogens indicated overall occurrences of 1% (1/104) for *A. phagocytophilum* in *H. elliptica,* 9.6% (10/104) of *Rickettsia* spp. in *H. elliptica* and *R. sanguineus*, 5.8% (6/104) of *Ehrlichia canis* in *H. elliptica* and *R. sanguineus*, and 13.5% (14/104) of *Coxiella* spp. in both *H. elliptica* and *R. sanguineus*. Additionally, PCR detected 6.5% (2/31) of *Coxiella* spp. DNA from *H. elliptica* eggs. Our data indicate that urban stray dogs admitted at PAWS are infested by *H. elliptica* and *R. sanguineus* ticks which are harbouring several pathogenic organisms known to cause tick-borne diseases.

## 1. Introduction

Ticks are blood feeding acarines with body sizes ranging from 2–30 mm infesting domestic and wild animals [1]. To date there are four described families of ticks including Ixodidae, Argasidae, Nuttalliellidae, and the extinct Deinocrotonidae [2,3,4]. Ticks are of great medical and veterinary significance due to their ability to transmit several pathogenic microorganisms to human and animal hosts [5,6]. These tick-borne pathogens are transmitted to other ticks and hosts associated with ticks by transovarial and transstadial transmission [7,8].

Ticks primarily feeding on companion animals may feed on humans in the absence of preferred hosts, resulting in the incidental transmission of tick-borne pathogens to humans [9]. In addition, these ticks are well adapted to urban environments, to the extent that female ticks deposit eggs in cracks of walls or inside dog bedding, resulting in tick infestations among human settlements [7]. Several tick-borne pathogens from ticks infesting dogs that are well-documented include *Anaplasma phagocytophilum* which is associated with human and canine granulocytic anaplasmosis, *Babesia* spp. causing canine babesiosis, *Coxiella* spp. causing Q-fever in humans, *Ehrlichia canis* associated with canine ehrlichiosis, as well as *Rickettsia* spp. causing African tick bite fever, Mediterranean spotted fever, and Astrakhan fever [7,10,11,12].

There is always a need to monitor and document ticks infesting urban dogs and their associated tick-borne pathogens due to their close association with human beings, particularly using modern DNA based techniques. This study aimed to identify ticks and their associated tick-borne pathogens, infesting stray dogs housed at the Potchefstroom Animal Welfare Society (PAWS) which originated from urban settlements. Ticks and tick-borne pathogens were identified by using a combination of morphological and molecular methods.

## 2. Results

### 2.1. Identification of Ticks

A total of 592 ticks were collected from 61 dogs admitted to PAWS originating from several suburbs in Potchefstroom (Table 1). These ticks were morphologically identified as *H. elliptica* (GTTM voucher accession number: OP5113) and *R. sanguineus* (GTTM voucher accession number: OP5078). The overall occurrence of *H. elliptica* was 39% (231/592), where *R. sanguineus* had an overall occurrence of 61% (361/592). In the respective species, 67.5% (156/231) and 48.5% (175/361) were female, and 32.5% (75/231) and 51.5% (186/361) were male. Of these ticks *H. elliptica* consisted of 43.7% (101/231) nymphs and 56.3% (130/231) adults, whereas *R. sanguineus* consisted of 60.9% (220/361) nymphs and 39.1% (141/361) adults. The *H. elliptica* species was most abundant in Miederpark (22.47%), whilst *R. sanguineus* was most abundant in both Miederpark (16.39%) and Ikageng (13.85%). There was a significant difference in tick species occurrence based on the geographical localities of *R. sanguineus* (X^2^ = 495.09, df = 9, *p*-value < 2.2 × 10^−16^) and *H. elliptica* (X^2^ = 629.3, df = 9, *p*-value < 2.2 × 10^−16^) ticks collected at various sampled sites.

The BLASTn search results of the *CO1* and *ITS2* genes indicated that *R. sanguineus* sequences of this study (GenBank accession numbers: MK295614, MK295616, MK295617, and MK295618) were similar to other *R. sanguineus* sequences on the NCBI database with matching identifications of 99% for the *CO1* gene and 94 to 97% for the *ITS2* gene. For *H. elliptica* there was no reference sequence available on the NCBI database. However, BLASTn search indicated *H. elliptica* sequences from this study (GenBank accession numbers: MK295612, MK295613, and MK295615) were similar to several species among *Haemaphysalis* with pairwise identity between 87 to 99% for *CO1*, and 85 to 96% for *ITS2*. Phylogenetic analysis of *CO1* (Supplementary Figure S1) and *ITS2* (Supplementary Figure S2) revealed the presence of two major clades. In one of these clades, all *Rhipicephalus* species clustered together and in the other clade all *Haemaphysalis* species clustered together.

### 2.2. Molecular Detection of Tick-Borne Pathogens

The PCR agarose gel images are shown in Supplementary Figures S3, S5, S7 and S9. Out of the 104 tick pools screened for the presence of tick-borne pathogens there was an overall occurrence of 1% (1/104) for *A. phagocytophilum,* 9.6% (10/104) for *Rickettsia*, 5.8% (6/104) for *Ehrlichia canis*, and 13.5% (14/104) for *Coxiella* spp. Table 2 represents the occurrence of several tick-borne pathogens of *H. elliptica* and *R. sanguineus* tick pools of dogs originating from several suburbs in Potchefstroom, as well as from the Fochville and Boipatong suburbs. Of the 31 egg pools screened for the presence of tick-borne pathogens, only *Coxiella* spp. was detected with an overall occurrence of 6.5% (2/31). These pathogens were detected in 1 of 2 (50%) and 1 of 11 (9.1%) *H. elliptica* egg batches of dogs originating from Boipatong and Miederpark suburbs, respectively.

*Anaplasma phagocytophilum* were detected in 2% (1/49) of *H. elliptica* ticks. There was no *A. phagocytophilum* detected from *R. sanguineus* tick DNA extracts. There was no significant statistical difference (X^2^ = 9, df = 9, *p*-value = 0.4373) of *A. phagocytophilum* prevalence based on geographical localities. The *E. canis* was detected in 8.2% (4/49) of *H. elliptica* ticks and 3.6% (2/55) of *R. sanguineus* ticks. There was a significant statistical difference (X^2^ = 37.333, df = 9, *p*-value = 2.295 × 10^−5^) in prevalence of *E. canis* based on the geographical localities. *Rickettsia* spp. were detected in 14.3% (7/49) of *H. elliptica* ticks and 5.5% (3/55) of *R. sanguineus* ticks with significant difference of prevalence (X^2^ = 32, df = 9, *p*-value = 0.0001991) when geographical localities were compared. *Coxiella* spp. was detected in 10.5% (2/19) of *H. elliptica* eggs, 22.4% (11/49) of *H. elliptica* ticks and 5.5% (3/55) of *R. sanguineus* ticks with a significant statistical difference (X^2^ = 60.286, df = 9, *p*-value = 1.181 × 10^−9^) in the geographical localities in ticks, whereas there was no significant statistical difference (X^2^ = 5, df = 6, *p*-value = 0.5438) of *Coxiella* spp. prevalence of the 19 *H. elliptica* tick egg batches analysed. In addition, mixed infections of *A. phagocytophilum* and *Rickettsia* spp. were detected from ticks of dogs originating from Boipatong suburb as well as mixed *Coxiella* spp., *E. canis*, and *Rickettsia* spp. from dogs originating from Miederpark suburb.

The BLASTn results of *16s rRNA* gene of *A. phagocytophilum* detected in this study (GenBank accession number: MK295611) confirmed that it matches with relevant species on the NCBI database (GenBank accession numbers: AY623650.1 and MF787270.1) with matching pairwise identity scores of 99% (Supplementary Figure S3).

The *gltA* gene results of *Rickettsia* from this study (GenBank accession numbers: MK295619, MK295620, MK295621, MK295622, MK295623, and MK295624) were similar to *Rickettsia conorii* sequences on the NCBI database (GenBank accession numbers: DQ821855.1, MF002509.1, and KY640399.1) with matching pairwise identity scores ranging between 98 and 100% (Supplementary Figure S4).

Similarly, the *16S rRNA* gene of *E. canis* detected in the current study were similar to *E. canis* sequences on the NCBI database (GenBank accession numbers: DQ494536.1, JQ976640.1, and MF153965.1) with matching pairwise identity scores ranging between 85 and 100% (Supplementary Figure S5).

Furthermore, the results of *IS1111 transposase* gene of *Coxiella* spp. of this study were similar to *Coxiella* spp. sequences on the NCBI database (GenBank accession numbers: JF970261.1, MH394636.1, and CP014563.1) with matching pairwise identity ranging between 95 and 100%, respectively (Supplementary Figure S6).

## 3. Discussion

In this study, *R. sanguineus* were identified as the most abundant tick species infesting dogs admitted to PAWS, in the North West Province, as compared to *H. elliptica*. This was expected as findings of previous studies [13,14,15,16] suggested that the most abundant species infesting South African companion animals are *R. sanguineus*, *H. elliptica*, and *R. simus*. Results of this study were similar to studies conducted by Mtshali [17] with reported occurrences of 49.9% and 5% of *R. sanguineus* and *H. elliptica,* respectively, collected from companion animals in Mafikeng, North West Province. Furthermore, Bryson et al. [16] reported respective occurrences of 96.62% and 2.85% for *R. sanguineus* and *H. elliptica* in the North West Province. In the province of Mpumalanga, Kolo et al. [18] reported 27 *R. sanguineus* and 30 *H. elliptica* from a total of 103 ticks infesting dogs. In this study, the most ticks were collected from dogs originating from Miederpark (where *H. elliptica* was more abundant than *R. sanguineus*) and Ikageng (where *R. sanguineus* was more abundant) suburbs of Potchefstroom. These collections were from stray dogs housed at PAWS which is located in Miederpark, whilst Ikageng is a settlement where many strays occur. The *R. sanguineus* ticks are behaviourally adapted to survive in urban settlements and dog kennels for extended time periods [6]. If preferred hosts are absent, these ticks will readily infest other hosts such as other domestic animals, livestock, and humans [16,19,20]. Rautenbach et al. [14], Bechara et al. [21] and Little et al. [19] suggested that *R. sanguineus* are reported in higher abundance in settlements where strays are present, or in animal shelters where dogs are co-housed, because tick control measures are lacking. The *H. elliptica* ticks prefer to infest murid rodents during the larval and nymphal developmental stages, whereas the adult stage prefers to infest members of the Canidae family [13,16,22]. Horak [15] and Bryson et al. [16] suggested that *H. elliptica* are often reported in higher abundance from communities with access to modern veterinary services. This might explain their lower abundances in other suburbs of Potchefstroom [13,16,22]. In addition, Chong et al. [23] and Lebert et al. [24] suggested that the difference in the distribution of the locations of different tick species, as well as the difference in their numbers in the different developmental stages may be due to several factors, including but not limited to seasonality of the tick species, differences in climatic conditions and availability of preferred hosts.

The *ITS2* and *CO1* genes were used to supplement morphological identification of tick species. A study conducted by Fukunaga et al. [25] revealed that the use of *ITS2* nucleotide sequences was able to distinguish between ticks sharing the same morphological features or synonymized tick species. The *CO1* gene is often used as a standard barcode for animal identification [26,27,28,29]. Lv et al. [29] suggested that the combined use of the *ITS2* and *CO1* gene, along with other genes, including *12S rDNA*, *16S rDNA*, and *18S rDNA*, give more reliable results.

Molecular detection of *Anaplasma* spp. detected in this study using species-specific primers was similar to observations made in previous studies conducted in South Africa. A study by Mtshali et al. [30] reported presence of *Anaplasma*-like organisms from dog and ticks. Inokuma et al. [31] also reported the presence of *Anaplasma* spp. infesting dogs from South Africa that are closely related to *A. phagocytophilum* and *A. platys*. The low occurrence may be due to *A. phagocytophilum* that activates cytopenias and reduces the amount of white and red blood cells which influences the infection ability of other haemoparasites [32].

*Rickettsia* species were detected from DNA of tick pools by PCR using genus specific *Rickettsia* primers. These were identified as *R. conorii* by sequencing. Fourie et al. [33] suggested that *R. conorii* infections in both *R. sanguineus* and *H. elliptica* ticks are possible. This pathogen has previously been detected in *R. sanguineus* ticks [34,35], and in *H. elliptica* [36]. Mtshali et al. [30] reported an overall occurrence of 38% for *R. conorii* and *R. africae* in dog ticks in the North West Province using PCR. In another study conducted by Kolo et al. [18], *Rickettsia* spp. infestation rates of 70% were reported from ticks and blood from dogs in the Mpumalanga province. Members of *Rickettsia* genus are known to be obligatory intracellular parasites or mutualists of arthropods [37] which explains their consistent positive detection from ticks. The European Food Safety Authority (EFSA) Panel on Animal Health and Welfare (AHAW) (2010) [38] and Uilenberg et al. [39] stated that although *R. conorii* mainly infect *R. sanguineus*, the transmission of this pathogen to humans is possible by *H. elliptica* as well. *Rickettsia* infections, due to tick bites from several species, were also reported by as well as from humans that either reside in or travelled to South Africa [40,41,42,43,44,45].

In the current study, *Babesia* spp. were not detected by the conventional PCR was used. Even though *Babesia* infections are commonly reported in companion animals in South Africa, infection rates seem to differ. Allan [46] reported the presence of *B. rossi* and *B. vogeli* with respective infection rates of 12.7% and 3.2% in Cape Town. Furthermore, the occurrence of *B. rossi* with respective infection rates of 75% and 32.1% and *B. vogeli* with 3% and 1.8% in dog blood was reported in the city of Pretoria, whilst these piroplasms were also detected from several tick species infesting dogs in the same city [15,16]. Schetters et al. [47], Horak [15] and Bryson et al. [16] suggested that although ticks are generally collected from dogs in South Africa which have been diagnosed with canine babesiosis, they are not essential vectors of this pathogen, possibly explaining lack of *Babesia* spp. detection in this study.

During the current study, the presence of *E. canis* was detected only by PCR from tick pools. Infections of *E. canis* in *R. sanguineus* ticks were previously reported by studies conducted by Murphy et al. [48], Aguiar et al. [49] and Harrus et al. [50]. Furthermore, *H. elliptica* are not recognised vectors of *E. canis*; however, Ogbu et al. [51] suggested that *E. canis* may be transmitted to other tick species after exposure to an infected host, possibly explaining the *E. canis* infection in *H. elliptica* observed during this study. Mtshali et al. [30] reported *E. canis* infections in the North West Province, whilst 16% were reported in Mpumalanga, 12.7% in Cape Town, 42% in Bloemfontein, and 17.2%, as well as 3% in Maboloka were reported by Kolo et al. [18], Allan [46], Pretorius and Kelly [52], Rautenbach et al. [14], and Matjila et al. [53], respectively. The *E. canis* infections are difficult to detect when using blood smears, due to low parasitaemia [14,52,54,55]. This may explain why this pathogen was absent in Giemsa-stained blood smears but could be detected by PCR.

*Coxiella* spp. was detected in the current study by PCR from tick pools as well as from egg batches. As suggested by Woldehiwet [56], de la Fuente et al. [11], as well as Angelakis and Raoult [57] *Coxiella* spp. may be transmitted by means of transstadial and transovarial transmission, thus suggesting the presence of *Coxiella* spp. in tick eggs as well as ticks during their different developmental stages. Mtshali et al. [30] reported an overall occurrence of 31% for *Coxiella* spp. from ticks infesting companion animals in the North West province. Duron et al. [58] also reported infections of Coxiella-like endosymbionts in several tick species, including *R. decoloratus* and *R. microplus* infesting wildlife in South Africa. Baca and Paretsky [59], Dupont et al. [40], Buhariwalla et al. [60], Zhang et al. [61], Loftis et al. [62] and Duron et al. [58] reported that *Coxiella* spp. infections in humans are quite common and globally reported, due to the inhalation of contaminated aerosol particles. This may be an indication that the majority of human infections are due to the association between humans and infected livestock. Heinzen et al. [63], Mediannikov et al. [64] and Duron et al. [58] suggests that arthropods, especially ticks, are not vital in maintaining transmission of *Coxiella* spp. to humans or other animals, but this pathogen may be transmitted by means of transstadial transmission during blood meals of infected ticks. This often results in Q-fever infections in reservoir hosts, including companion animals, and accidental hosts, including humans.

The study indicated mixed infections of several tick-borne pathogens. These include mixed infections of *A. phagocytophilum* and *R. conorii*, as well as *R. conorii*, *E. canis*, and *Coxiella* spp. Van Heerden [65] and Pennisi et al. [66] suggested that mixed infections are common, especially where domestic animals are co-housed. Matjila et al. [53,67] suggested that mixed infections may be attributed to *R. sanguineus* and *H. elliptica* ticks feeding on similar infected hosts in overlapping regions. Griffiths et al. [68] suggested that mixed infections are significant as pathogens present within the host interact with one another. These interactions may improve the transmission and progression of the associated diseases or cause disturbances in the colonization or virulence of other pathogens. Even though ticks are well known for their ability to transmit pathogenic organisms to their hosts the detection of medically and veterinary important tick-borne pathogens, associated with companion animals, in this study and previous studies [14,15,16,18,30,31,34,35,36,46,48,49,50,52,58,67] raises concern. These animal hosts often suffer due to illness caused by the pathogens. In several cases, infection of tick-borne pathogens may result in host mortality [20,69]. Several species of Ixodid ticks follow a three-host life cycle. This requires a blood meal during each developmental stage, of the ticks, from various hosts to enable life cycle completion. In the absence of companion animals, the ticks may feed on alternative hosts, including humans, resulting in zoonosis [11,16].

## 4. Materials and Methods

### 4.1. Sampling and Areas of Origin for the Dogs

**Figure 1:**
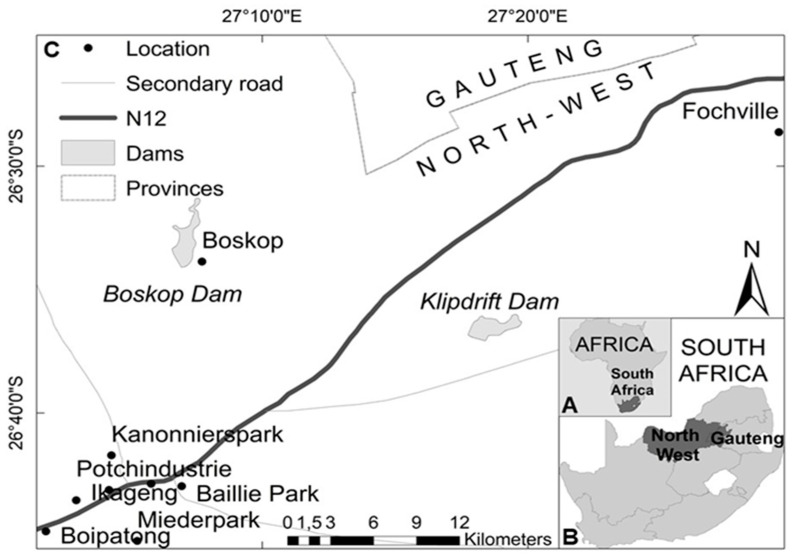
Maps indicating the sampling locations (made with ARGIS). (**A**) indicates a map of Africa showing South Africa. (**B**) indicates a map of South Africa showing the different sampling provinces. (**C**) indicates the locations of origin of sampled dogs.

Tick specimens (N = 592) were collected by the veterinarian from rescued stray dogs (N = 61) on arrival for admission at PAWS in 2017–2020 (Table 1). The suburbs where dogs originated from before they were admitted to PAWS are Potchindustrie (26°43′7.37″ S, 27°4′14.0952″ E), Boskop (26°33′51.9836″ S, 27°7′44.0036″ E), Kanonnierspark (26°41′43.0631″ S, 27°4′19.3904″ E), Baillie Park (26°42′58.1173″ S, 27°6′58.2487″ E), Ikageng (26°43′32.3738″ S, 27°2′59.9492″ E), Die Bult (26°42′52.3069″ S, 27°5′49.371″ E), Miederpark (26°45′11.362″ S, 27°5′17.6428″ E), Boipatong (26°44′48.0476″ S, 27°1′51.492″ E) and Fochville (26°28′37.4059″ S, 27°29′27.1457″ E) (Figure 1).

### 4.2. Morphological Identification of Ticks by Microscopy

Tick specimens were collected weekly from stray dogs from urban areas which were admitted to PAWS. Ticks were morphologically identified to species level by using the Nikon SMZ745 stereo microscope and identification keys of Horak et al. [70], Barker and Walker [71] and Walker et al. [72]. To confirm correct morphological identification, representatives of each tick species were submitted to the Gertrud Theiler Tick Museum, located at the Agricultural Research Council-Onderstepoort Veterinary Research (ARC-OVR) and voucher numbers were issued. All of the engorged female ticks (N = 204) collected during the study were kept, while they were still alive, in separate containers until eggs were laid. Afterwards the dead ticks and eggs were stored in 70% ethanol for a different study, however, the eggs were included for molecular detection of tick-borne pathogens.

### 4.3. Molecular Identification of Ticks

In preparation for molecular identification of the tick species, legs were removed from selected tick samples for DNA extraction. For molecular detection of tick-borne pathogens, ticks were pooled together according to the same species, host, the hosts location of origin, and life stage, from which they were collected. Tick pools consisted of three or four ticks, however, in cases where there were less than three specimens, the samples were not pooled but were treated separately as individual samples. Additionally, for the molecular detection of tick-borne pathogens, the tick egg batches (each made up of 50 eggs) originating from the same tick species and location were stored. In total there were 104 tick pools and 31 egg batches. Prior to DNA extraction, ticks and eggs were surface sterilized for 1 h with 10% Tween 20 and then rinsed twice with 70% ethanol and rinsed three times with double distilled water. Genomic DNA (gDNA) was extracted from legs, tick pools, and egg batches by the salting out method as described by Riveroa et al. [73] and stored at −35 °C until further use.

The cytochrome oxidase subunit 1 (*CO1*) and internal transcribed spacer 2 (*ITS2*) were the targeted gene regions for molecular identification of the collected tick samples. The PCR for the amplification of the *CO1* gene was conducted using primers LCO1490 forward (GGT CAA CAA ATC ATA AAG ATA TTG G) and HCO2198 reverse (TAA ACT TCA GGG TGA CCA AAA AAT CA), and the *ITS2* gene using primers ITS2F forward (YTG CGA RAC TTG GTG TGA AT) and ITS2R reverse (TAT GCT TAA RTT YAG SGG GT) described by Licari et al. [74] and Muruthi [3], respectively. For both gene regions, the PCR reaction mixture had a final volume of 25 µL which consisted of 12.5 μL of AmpliTaq Gold 360^®^ Master Mix (Applied Biosystems, Woodlands, Singapore), 1 μL each of primer [each at 10 μM concentration], 2 μL of the template DNA, and 8.5 μL double distilled water. *Haemaphysalis longicornis* DNA (obtained from Obihiro University of Agriculture and Veterinary Medicine, Obihiro, Japan) was used as positive control, while distilled water was used as negative control. PCR conditions consisted of initial denaturation at 95 °C for 10 min, 35 cycles of denaturation at 95 °C for 30 s, annealing at 47 °C (*CO1* gene) and 50 °C (*ITS2* gene) for 30 s, and extension at 72 °C for 60 s, followed by a final extension at 72 °C for 7 min and final hold at 4 °C, using the ProFlex PCR System (Applied Biosystems, Woodlands, Singapore).

### 4.4. Molecular Detection of Tick-Borne Pathogens

Molecular detection of tick-borne pathogens, namely *Anaplasma phagocytophilum*, *Babesia* spp., *Coxiella* spp., *Rickettsia* spp. and *Ehrlichia canis* from 104 tick pools and 31 egg batches were analysed where the PCR mixture was prepared as described above using species specific PCR primers for the different pathogens. The PCR was conducted targeting the *16S rRNA* gene to determine the presence of *A. phagocytophilum* [75] and *E. canis* [76] by, respectively, using the EHR-521 forward (TGT AGG CGG TTC GGT AAG TTA AAG) and EHR-747 reverse (GCA CTC ATC GTT TAC AGG GTG) primers, as well as the E.c 16S forward (TCG CTA TTA GAT GAG CCT ACG T) and E.c 16S reverse (GAG TCT GGA CCG TAT CTC AGT) primers. For *Babesia* spp., the *18S rRNA* gene (Duarte et al., 2008) was targeted for the detection of *B. canis*, using the primers BAB1 forward (GTG AAC CTT ATC ACT TAA AGG) and BAB3 reverse (CTA CAC AGA GCA CAC AGC C), *B. vogeli*, using the primers BAB1 forward (GTG AAC CTT ATC ACT TAA AGG) and BAB4 reverse (CAA CTC CTC CAC GCA ATC G), as well as *B. rossi,* using the primers BAB1 forward (GTG AAC CTT ATC ACT TAA AGG) and BAB5 reverse (AGG AGT TGC TTA CGC ACT CA). For the detection of *Rickettsia* spp., the *gltA* gene [77] was targeted using the primers Rp877p forward (GGG GAC CTG CTC ACG GCG G) and Rp1258n reverse (ATT GCA AAA AGT ACA GTG AAC A). The detection of *Coxiella* spp. was performed targeting the *IS1111 transposase* gene [78] using the primers IS1111aF forward (CAT CAC ATT GCC GCG TTT AC) and IS1111aR reverse (GGT TGG TCC CTC GAC AAC AT). For positive controls, *A. phagocytophilum* DNA was obtained from a PCR positive horse from Northern Cape [79] while controls of *E. canis, R. africae* and *Coxiella* spp. DNA acquired from the Research Centre for Zoonosis Control, Hokkaido University, Japan were used. *Babesia canis* positive controls were synthesized from gBlock^®^ gene fragments obtained from Whitehead Scientific (Pty) Ltd., Cape Town, South Africa. The PCR conditions consisted of initial denaturation at 95 °C for 30 s, 30 cycles of denaturation at 95 °C for 30 s, annealing at 50 °C (*B. canis*, *B. rossi*, and *B. vogeli*), 52 °C (*Rickettsia* spp.), 57 °C (*Coxiella* spp.), and 60 °C (*A. phagocytophilum* and *E. canis*) for 60 s and extension at 68 °C for 60 s followed by a final extension at 68 °C for 5 min and final hold at 4 °C, using the ProFlex PCR System (Applied Biosystems, Woodlands, Singapore).

The PCR products were purified using the QIAquick Gel Extraction Kit (Qiagen, DE, Hilden, Germany) by following the manufactures instructions (Qiagen, DE, Hilden, Germany). Purified PCR products were submitted for sequencing at Inqaba Biotechnological Industries (Pty) Ltd., Pretoria, South Africa. Sequence visualisation and editing was performed by using Molecular Evolutionary Genetics Analysis version 7.0 (MEGA7) software package [14]. The nucleotide Basic Local Alignment Search Tool (BLASTn) was used to confirm tick and tick-borne pathogen identification (https://blast.ncbi.nlm.nih.gov/Blast.cgi, accessed on 15 August 2017).

### 4.5. Blood Smears

Giemsa stained thin blood smears were prepared of engorged ticks by using a modified method described by Poostchi et al. [80].

### 4.6. Statistical Analysis

The significance relating to the geographic localities of ticks as well as tick-borne pathogens was determined in R-studio by using the Pearson’s chi-square test. Confidence interval (CI) of an average of 95% was used to determine tick and tick-borne pathogens occurrences. Phylogenetic trees were constructed using *CO1* and *ITS2* gene sequences obtained from this study, along with homologous sequences of closely related species obtained from the NCBI database. All sequences were added to the alignment explorer in MEGA7, aligned by ClustalW using default parameters and trimmed to be even length. Lowest Bayesian Information Criterion (BIC) score was used to determine the best nucleotide substitution model. Maximum likelihood method was used for construction of the phylogenetic trees with 10,000 bootstrap replications. During phylogenetic analysis, missing nucleotide data or gaps were removed and rates among sites were handled as uniform rates.

## 5. Conclusions

The ticks collected in this study were identified as *R. sanguineus* and *H. elliptica* in accordance with other studies and the literature. This is an indication that ticks flourish in environments where stray dogs are present, especially in the absence of tick control measures. This study also demonstrated the presence of tick-borne pathogens including, *A. phagocytophilum*, *R. conorii*, *E. canis*, and *Coxiella* spp., in ticks and their eggs indicating a cause for concern with regards to the health of companion animals and humans as most of these species are associated with zoonotic diseases.

## Figures and Tables

**Table 1:** Tick abundance from sampled dogs at PAWS and their various locations.

Location	Tick Species		Total Number of Ticks per Location	Total Number of Dogs per Location
	*H. elliptica* (%) ^a^	*R. sanguineus* (%) ^a^		
Potchefstroom **^b^**	4 (3.31)	117 (96.69)	121	7
Potchindustrie	7 (21.88)	25 (78.13)	32	2
Boskop	21 (77.78)	6 (22.22)	27	4
Die Bult	11 (57.89)	8 (42.11)	19	6
Baillie park	4 (18.18)	18 (81.82)	22	3
Miederpark	133 (57.83)	97 (42.17)	230	27
Boipatong **^c^**	38 (88.37)	5 (11.63)	43	4
Ikageng	4 (4.65)	82 (95.35)	86	6
Fochville **^c^**	9 (100)	-	9	1
Kannonierspark	-	3 (100)	3	1
Total number of ticks	231	361	592	61

**^a^**: Indicates the occurrence of ticks from dogs originating from several locations in percentages; **^b^**: Ticks collected from dogs originating from Potchefstroom, but their exact locations of origin was unknown; **^c^**: Incidental tick samples from dogs not originating from Potchefstroom although admitted at PAWS Potchefstroom.

**Table 2:** Overall occurrence of tick-borne pathogens detected by PCR from tick pools.

Location	Species	*A. phagocytophilum* (%) ^a^	*Rickettsia* sp. (%) ^a^	*B. canis* (%) ^a^	*B. vogeli* (%) ^a^	*B. rossi* (%) ^a^	*E. canis* (%) ^a^	*Coxiella* spp. (%) ^a^	Total Pools Screened
Baillie park	*H. elliptica*	-	-	-	-	-	-	-	1
	*R. sanguineus*	-	2 (50)	-	-	-	-	1 (25)	4
Boipatong ^**b**^	*H. elliptica*	1 (33.3)	1 (33.3)	-	-	-	-	-	3
	*R. sanguineus*	-	-	-	-	-	-	-	3
Die Bult	*H. elliptica*	-	-	-	-	-	-	-	4
	*R. sanguineus*	-	-	-	-	-	-	-	3
Fochville ^**b**^	*H. elliptica*	-	-	-	-	-	-	1 (50)	2
Ikageng	*H. elliptica*	-	-	-	-	-	-	-	4
	*R. sanguineus*	-	-	-	-	-	-	-	10
Kannonierspark	*R. sanguineus*	-	-	-	-	-	-	1 (50)	2
Miederpark	*H. elliptica*	-	6 (24)	-	-	-	4 (16)	9 (36)	25
	*R. sanguineus*	-	-	-	-	-	1 (4.3)	1 (4.3)	23
Boskop	*H. elliptica*	-	-	-	-	-	-	1 (20)	5
	*R. sanguineus*	-	-	-	-	-	1 (50)	-	2
Potchindustrie	*H. elliptica*	-	-	-	-	-	-	-	1
	*R. sanguineus*	-	-	-	-	-	-	-	2
Potchefstroom ^**c**^	*H. elliptica*	-	-	-	-	-	-	-	4
	*R. sanguineus*	-	1 (16.7)	-	-	-	-	-	6
**Total**		**1 (1)**	**10 (9.6)**	**0 (0)**	**0 (0)**	**0 (0)**	**6 (5.8)**	**14 (13.5)**	**104**

**a**: Indicates the occurrence of ticks from dogs originating from several locations in percentages; **b**: incidental tick samples from dogs not originating from Potchefstroom; **c**: Ticks collected from dogs originating from Potchefstroom, but their exact locations of origin was unknown.

## Data Availability

Not applicable.

## Supplementary Materials

The following are available online at https://www.mdpi.com/article/10.3390/pathogens11080862/s1. Figure S1. Phylogenetic analysis of tick *CO1* gene sequences using the Maximum Likelihood (ML) method based on the General Time Reversible (NTR) model [81]. Bootstrap percentage of 10,000 replicates, in which the associated taxa are clustered together is displayed next to the branch nodes. Twelve nucleotide sequences were used for data analysis. Sequences of this study are indicated by a black bullet. *Myialges* spp. was used as an outgroup. Phylogenetic analysis was performed by using MEGA 7 [82]. Figure S2. Phylogenetic analysis of tick ITS2 gene using the Maximum Likelihood (ML) method based on the Kimura 2-parameter model [83]. Bootstrap percentage of 10,000 replicates, in which the associated taxa are clustered together is displayed next to the branch nodes. Fourteen nucleotide sequences were used for data analysis. Sequences of this study are indicated by a black bullet. *Dermanyssus* sp. was used as an outgroup. Phylogenetic analysis was performed by using MEGA 7 [81]. Figure S3: Fragment of the BLASTn alignment between *A. phagocytophilum* of this study and a corresponding sequence. First strand represents *A. phagocytophilum* detected from ticks collected from the JB Marks local municipality. Second strand represents a reference sequence from NCBI. Red arrows indicate where nucleotides mismatch. Figure S4: Fragment of the BLASTn alignment between R. conorii of this study and a corresponding sequence. First strand represents R. conorii detected from ticks collected from the JB Marks local municipality. Second strand represents a reference sequence from NCBI. Red arrows indicate where nucleotides mismatch. Figure S5: Fragment of the BLASTn alignment between E. canis of this study and a corresponding sequence. First strand represents E. canis detected from ticks collected from the JB Marks local municipality. Second strand represents a reference sequence from NCBI. Red arrows indicate where nucleotides mismatch. Figure S6: Fragment of the BLASTn alignment between C. burnetii of this study and a corresponding sequence. First strand represents C. burnetii detected from ticks collected from the JB Marks local municipality. Second strand represents a reference sequence from NCBI
