# Supplementary material for: Detection of Ticks and Tick-Borne Pathogens of Urban Stray Dogs in South Africa
Source: Pathogens. 2022 Jul 30;11(8):862. doi: 10.3390/pathogens11080862 (PMC9416273; doi:10.3390/pathogens11080862)
Supplement: Supplementary file 1 [file pathogens-11-00862-s001.zip › pathogens-1802956-supplementary.pdf]

Supplementary files

Detection of ticks and tick-borne pathogens of urban stray dogs in South Africa

Clara-Lee van Wyk [1], Khethiwe Mtshali [2], Moeti O. Taioe [1,3], Stallone Terera [4], Deon Bakkes [5], Tsepo Ramatla [1], Xuenan Xuan[6,*] and Oriel Thekisoe [1,]

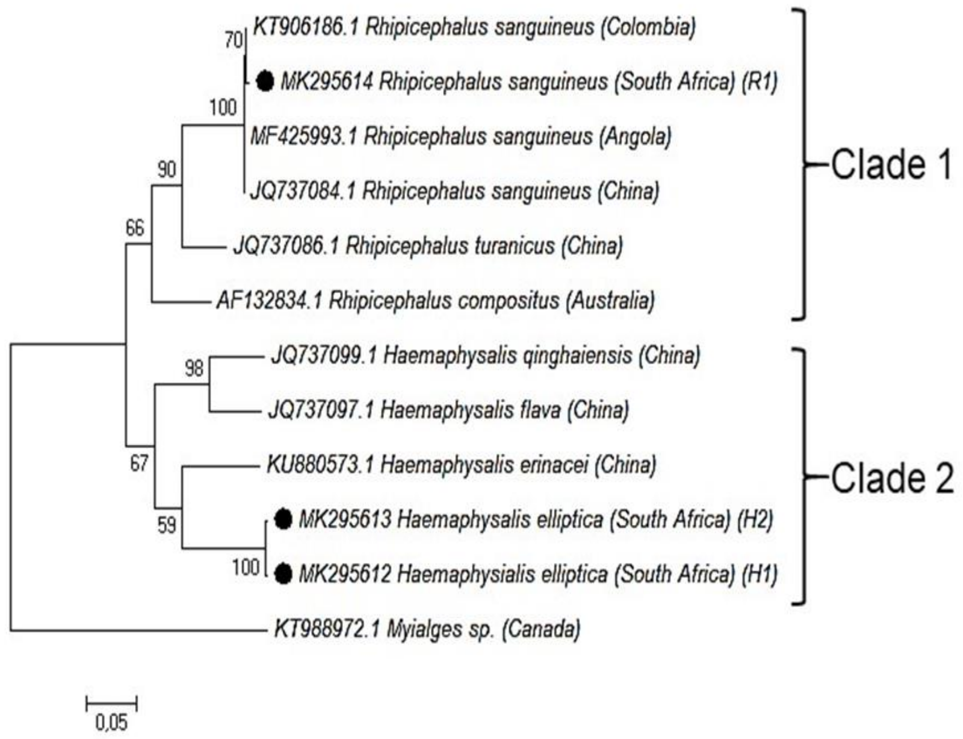

Figure S1. Phylogenetic analysis of tick *CO1* gene sequences using the Maximum Likelihood (ML) method based on the General Time Reversible (NTR) model [81]. Bootstrap percentage of 10 000 replicates, in which the associated taxa are clustered together is displayed next to the branch nodes. Twelve nucleotide sequences were used for data analysis. Sequences of this study are indicated by a black bullet. *Myialges* spp. was used as an outgroup. Phylogenetic analysis was done by using MEGA 7 [82]

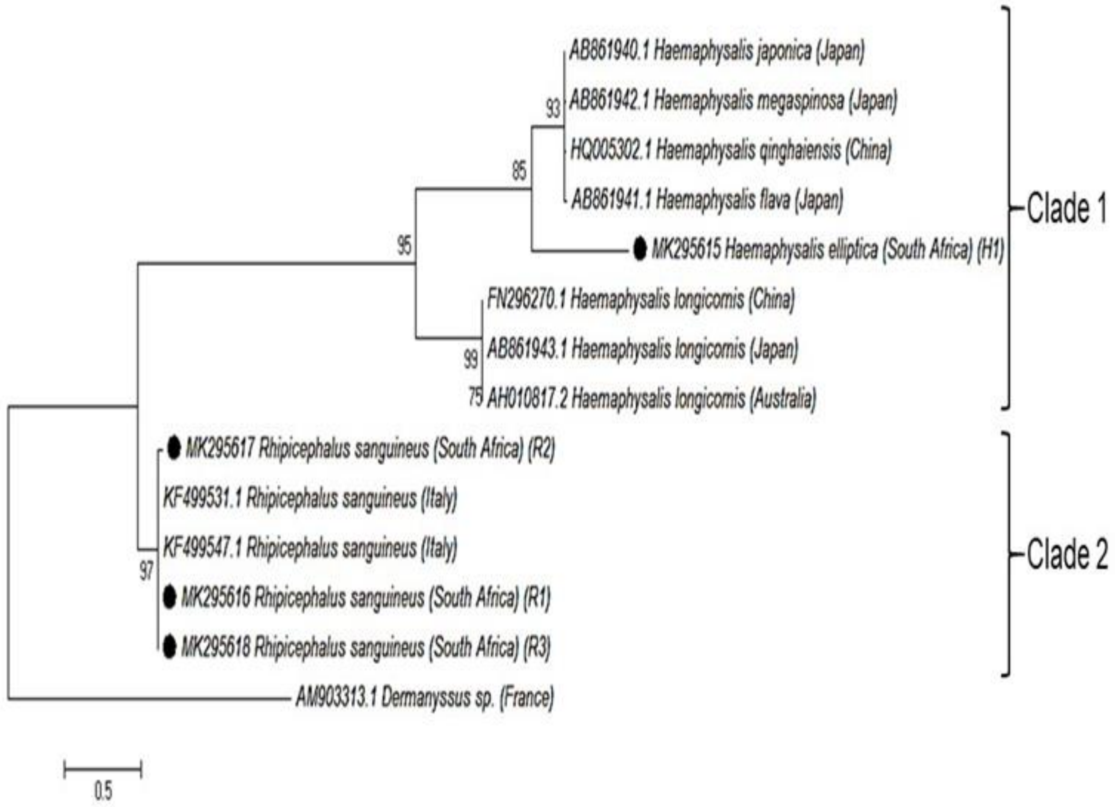

Figure S2. Phylogenetic analysis of tick ITS2 gene using the Maximum Likelihood (ML) method based on the Kimura 2-parameter model [83]. Bootstrap percentage of 10 000 replicates, in which the associated taxa are clustered together is displayed next to the branch nodes. Fourteen nucleotide sequences were used for data analysis. Sequences of this study are indicated by a black bullet. *Dermanyssus* sp. was used as an outgroup. Phylogenetic analysis was done by using MEGA 7 [82].

Sample ID	Gene size (bp)	BLAST description	Maximum score	Total score	Query cover	E-value	Identity	Accession number
BHA34	205	*Anaplasma phagocytophilum*	351	351	98%	6,00E-93	99%	AY623650.1
		Anaplasma phagocytophilum	349	349	96%	2,00E-92	99%	MF787270.1
		Anaplasma phagocytophilum	349	349	96%	2,00E-92	99%	MF787269.1
		Anaplasma phagocytophilum	349	349	96%	2,00E-92	99%	KX810088.1
		Anaplasma phagocytophilum	349	349	96%	2,00E-92	99%	KP062963.1

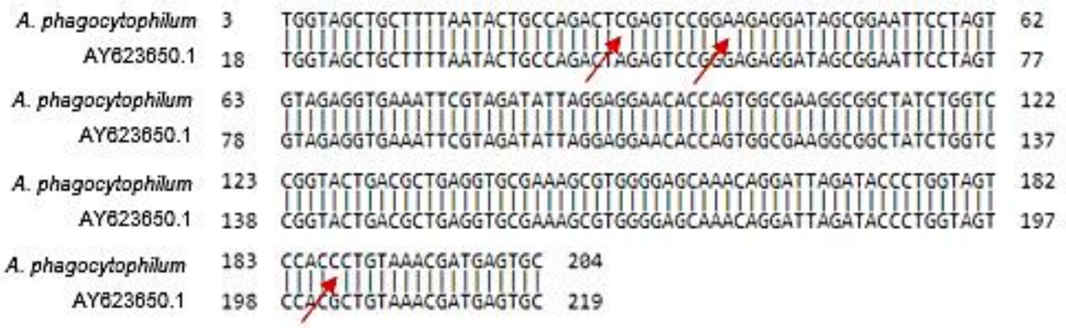

Figure S3: Fragment of the BLASTn alignment between *A. phagocytophilum* of this study and a corresponding sequence. First strand represents *A. phagocytophilum* detected from ticks collected from the JB Marks local municipality. Second strand represents a reference sequence from NCBI. Red arrows indicate where nucleotides mismatch

Sample ID	Gene size (bp)	BLAST description	Maximum score	Total score	Query cover	E-value	Identity	Accession number
BHA34	347	*Rickettsia conorii*	618	618	99%	5,00E-173	99%	DQ821855.1
		Rickettsia conorii	614	614	99%	6,00E-172	99%	MF002509.1
		Rickettsia conorii	614	614	99%	6,00E-172	99%	KY069261.1
		Rickettsia conorii	614	614	99%	6,00E-172	99%	KY640399.1
		Rickettsia conorii	614	614	99%	6,00E-172	99%	AE006914.1
BHA35	342	*Rickettsia conorii*	616	616	99%	2,00E-172	100%	DQ821855.1
		Rickettsia conorii	610	610	99%	7,00E-171	99%	MF002509.1
		Rickettsia conorii	610	610	99%	7,00E-171	99%	KY069261.1
		Rickettsia conorii	610	610	99%	7,00E-171	99%	KY640399.1
		Rickettsia conorii	610	610	99%	7,00E-171	99%	AE006914.1
BHA36B	348	*Rickettsia conorii*	612	612	99%	2,00E-171	99%	DQ821855.1
		Rickettsia conorii	607	607	99%	9,00E-170	99%	MF002509.1
		Rickettsia conorii	607	607	99%	9,00E-170	99%	KY069261.1
		Rickettsia conorii	607	607	99%	9,00E-170	99%	KY640399.1
		Rickettsia conorii	607	607	99%	9,00E-170	99%	AE006914.1
BHN36	346	*Rickettsia conorii*	612	612	99%	2,00E-171	99%	DQ821855.1
		Rickettsia conorii	609	609	99%	3,00E-170	99%	MF002509.1
		Rickettsia conorii	609	609	99%	3,00E-170	99%	KY069261.1
		Rickettsia conorii	609	609	99%	3,00E-170	99%	KY640399.1
		Rickettsia conorii	609	609	99%	3,00E-170	99%	AE006914.1
BRN45	395	*Rickettsia conorii*	569	569	93%	2,00E-158	98%	DQ821855.1
		Rickettsia conorii	567	567	94%	8,00E-158	98%	KY640399.1
		Rickettsia conorii	567	567	93%	8,00E-158	98%	AB872795.1

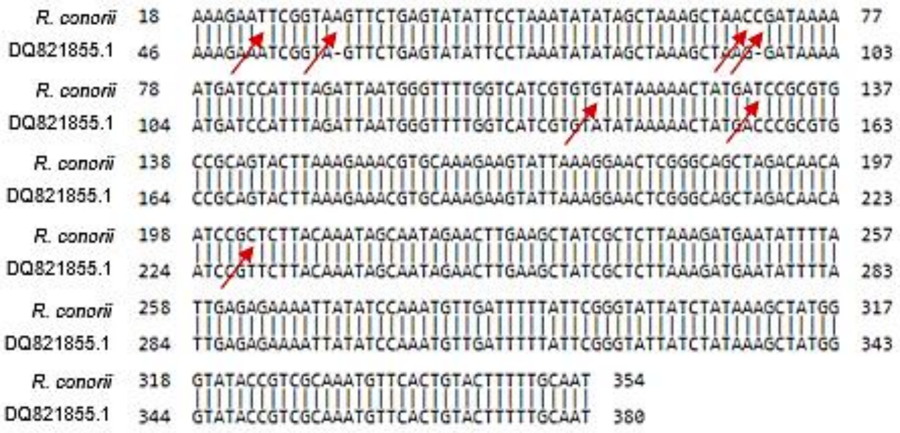

Figure S4: Fragment of the BLASTn alignment between *R. conorii* of this study and a corresponding sequence. First strand represents *R. conorii* detected from ticks collected from the JB Marks local municipality. Second strand represents a reference sequence from NCBI. Red arrows indicate where nucleotides mismatch

Sample ID	Gene size (bp)	BLAST description	Maximum score	Total score	Query cover	E-value	Identity	Accession number
BHA24	97	*Ehrlichia canis*	98,7	98,7	75%	7,00E-22	90%	DQ494536.1
		Ehrlichia canis	93,3	93,3	87%	3,00E-20	86%	MF059353.1
		Ehrlichia canis	93,3	93,3	62%	3,00E-20	93%	MF153971.1
		Ehrlichia canis	93,3	93,3	87%	3,00E-20	85%	DQ494537.1
		Ehrlichia canis	91,5	91,5	76%	1,00E-19	88%	JQ976640.1
BHA36B	99	*Ehrlichia canis*	120	120	86%	2,00E-28	91%	MF153965.1
		Ehrlichia canis	120	120	84%	2,00E-28	92%	JQ976640.1
		Ehrlichia canis	120	120	84%	2,00E-28	92%	JQ976631.1
		Ehrlichia canis	120	120	84%	2,00E-28	92%	DQ494536.1
		Ehrlichia canis	118	118	83%	8,00E-28	92%	KY594915.1
BRN16	89	*Ehrlichia canis*	156	156	96%	3,00E-39	100%	JQ976640.1
		Ehrlichia canis	156	156	96%	3,00E-39	100%	JQ976631.1
		Ehrlichia canis	154	154	95%	9,00E-39	100%	KY594915.1
		Ehrlichia canis	154	154	95%	9,00E-39	100%	CP025749.1
		Ehrlichia canis	154	154	95%	9,00E-39	100%	KX766395.1
BRN42	100	*Ehrlichia canis*	147	147	81%	2,00E-36	100%	MF059353.1
		Ehrlichia canis	145	145	80%	6,00E-36	100%	JQ976640.1
		Ehrlichia canis	145	145	80%	6,00E-36	100%	JQ976631.1
		Ehrlichia canis	143	143	79%	2,00E-35	100%	KY594915.1
		Ehrlichia canis	143	143	79%	2,00E-35	100%	CP025749.1

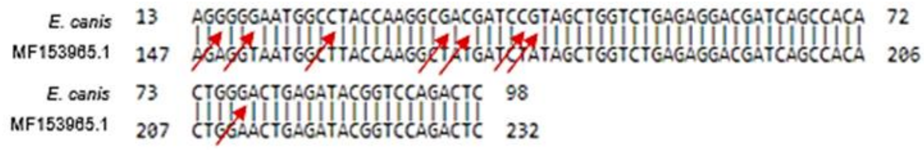

Figure S5: Fragment of the BLASTn alignment between *E. canis* of this study and a corresponding sequence. First strand represents *E. canis* detected from ticks collected from the JB Marks local municipality. Second strand represents a reference sequence from NCBI. Red arrows indicate where nucleotides mismatch

Sample ID	Gene size (bp)	BLAST description	Maximum score	Total score	Query cover	E-value	Identity	Accession number
BHN36	41	*Coxiella burnetii*	66,2	66,2	87%	2,00E-08	100%	JF970261.1
		Coxiella burnetii	62,6	62,6	82%	2,00E-07	100%	MH394636.1
		Coxiella burnetii	62,6	62,6	82%	2,00E-07	100%	CP014563.1
		Coxiella burnetii	62,6	62,6	82%	2,00E-07	100%	CP014561.1
		Coxiella burnetii	62,6	62,6	82%	2,00E-07	100%	CP014559.1
BHN37B	39	*Coxiella burnetii*	68	68	94%	4,00E-09	100%	MH394636.1
		Coxiella burnetii	68	68	94%	4,00E-09	100%	CP014563.1
		Coxiella burnetii	68	68	94%	4,00E-09	100%	CP014561.1
		Coxiella burnetii	68	68	94%	4,00E-09	100%	CP014559.1
		Coxiella burnetii	68	204	94%	4,00E-09	100%	CP014557.1
BHN42	38	*Coxiella burnetii*	57,2	57,2	92%	7,00E-06	97%	JF970261.1
		Coxiella burnetii	55,4	55,4	100%	2,00E-05	95%	MH394636.1
		Coxiella burnetii	55,4	55,4	100%	2,00E-05	95%	CP014563.1
		Coxiella burnetii	55,4	55,4	100%	2,00E-05	95%	CP014561.1
EH38	37	*Coxiella burnetii*	57,2	57,2	94%	6,00E-06	97%	MH394636.1
		Coxiella burnetii	57,2	57,2	94%	6,00E-06	97%	CP014563.1
		Coxiella burnetii	57,2	57,2	94%	6,00E-06	97%	CP014561.1
		Coxiella burnetii	57,2	57,2	94%	6,00E-06	97%	CP014559.1
		Coxiella burnetii	57,2	171	94%	6,00E-06	97%	CP014557.1

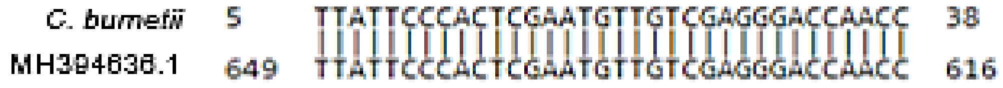

Figure S6: Fragment of the BLASTn alignment between *C. burnetii* of this study and a corresponding sequence. First strand represents *C. burnetii* detected from ticks collected from the JB Marks local municipality. Second strand represents a reference sequence from NCBI

References

81 Nei, M.; Kumar, S. Molecular Evolution and Phylogenetics New York: Oxford University Press; 2000. *Google Scholar*, **2000**, 87-103.

82 Kumar, S.; Stecher, G.; Tamura, K.; MEGA7: Molecular Evolutionary Genetics Analysis version 7.0 for bigger datasets. *Mol Biol Evol*. **2016**, 33, 1870-1874. https://doi.org/10.1093/molbev/msw054

83 Kimura, M. A simple method for estimating evolutionary rate of base substitutions through comparative studies of nucleotide sequences. *J. Mol. Evol*. **1980**, 16, 111-120. https://doi.org/10.1007/BF01731581
